# The efficacy of gait rehabilitations for the treatment of incomplete spinal cord injury: a systematic review and network meta-analysis

**DOI:** 10.1186/s13018-022-03459-w

**Published:** 2023-01-23

**Authors:** Tanyaporn Patathong, Krongkaew Klaewkasikum, Patarawan Woratanarat, Sasivimol Rattanasiri, Thunyarat Anothaisintawee, Thira Woratanarat, Ammarin Thakkinstian

**Affiliations:** 1 Department of Orthopedics, Faculty of Medicine Ramathibodi Hospital, Mahidol University, 270 Rama VI Road, Payathai, Ratchathewi, Bangkok, 10400 Thailand; 2 Department of Clinical Epidemiology and Biostatistics, Faculty of Medicine Ramathibodi Hospital, Mahidol University, Bangkok, 10400 Thailand; 3 Department of Family Medicine, Faculty of Medicine Ramathibodi Hospital, Mahidol University, Bangkok, 10400 Thailand; 4 Department of Preventive and Social Medicine, Faculty of Medicine, Chulalongkorn University, Bangkok, 10330 Thailand

**Keywords:** Functional electrical stimulation, Physical therapy, Robotic-assisted gait training, Treadmill, Walking

## Abstract

**Background:**

Recent pieces of evidence about the efficacy of gait rehabilitation for incomplete spinal cord injury remain unclear. We aimed to estimate the treatment effect and find the best gait rehabilitation to regain velocity, distance, and Walking Index Spinal Cord Injury (WISCI) among incomplete spinal cord injury patients.

**Method:**

PubMed and Scopus databases were searched from inception to October 2022. Randomized controlled trials (RCTs) were included in comparison with any of the following: conventional physical therapy, treadmill, functional electrical stimulation and robotic-assisted gait training, and reported at least one outcome. Two reviewers independently selected the studies and extracted the data. Meta-analysis was performed using random-effects or fixed-effect model according to the heterogeneity. Network meta-analysis (NMA) was indirectly compared with all interventions and reported as pooled unstandardized mean difference (USMD) and 95% confidence interval (CI). Surface under the cumulative ranking curve (SUCRA) was calculated to identify the best intervention.

**Results:**

We included 17 RCTs (709 participants) with the mean age of 43.9 years. Acute-phase robotic-assisted gait training significantly improved the velocity (USMD 0.1 m/s, 95% CI 0.05, 0.14), distance (USMD 64.75 m, 95% CI 27.24, 102.27), and WISCI (USMD 3.28, 95% CI 0.12, 6.45) compared to conventional physical therapy. In NMA, functional electrical stimulation had the highest probability of being the best intervention for velocity (66.6%, SUCRA 82.1) and distance (39.7%, SUCRA 67.4), followed by treadmill, functional electrical stimulation plus treadmill, robotic-assisted gait training, and conventional physical therapy, respectively.

**Conclusion:**

Functional electrical stimulation seems to be the best treatment to improve walking velocity and distance for incomplete spinal cord injury patients. However, a large-scale RCT is required to study the adverse events of these interventions.

*Trial registration*: PROSPERO number CRD42019145797.

**Supplementary Information:**

The online version contains supplementary material available at 10.1186/s13018-022-03459-w.

## Introduction

Spinal cord injury temporarily or permanently impairs automatic nervous system, motor, and sensory nerve conduction [1]. Its incidence ranges from 440 to 526 per million population [2]. Levels, types, and extent of injury reflect the muscle strength, spasticity, and gait pattern [3, 4]. Gait rehabilitation may help the patients, especially those with incomplete spinal cord injury, to regain their functions and walking ability [5–8]. Nowadays, widely used gait improvement programs [5, 9, 10] are hands-on therapy, overground gait training, exercises, and stretching maneuvers. Other potential methods include treadmill; the body-weight-supported treadmill training; functional electrical stimulation; and robotic-assisted gait training. Their possible benefits over conventional physical therapy were neuronal coordination, muscle strength, motor function, balance, walking, and physiologic gait improvement [5, 9, 11].

The efficacy of various gait rehabilitations has been evaluated. Conventional physical therapy insignificantly enhanced the gait velocity compared to the body-weight-supported treadmill training with or without electrical stimulation [12]. On the other hand, robotic-assisted gait training improved Walking Index of Spinal Cord Injury (WISCI) more than conventional physical therapy and treadmill [11, 13]. Evidence is still limited for functional electrical stimulation compared to other methods [14–16], particularly robotic-assisted gait training [7, 17]. Moreover, there is no comprehensive review of gait training interventions focused on walking ability (i.e., velocity and distance) and safety among incomplete spinal cord injury [11]. A network meta-analysis would be able to demonstrate myriad effects of these interventions.

This systematic review and network meta-analysis of randomized controlled trials (RCTs) aimed to find the best intervention for incomplete spinal cord injury, such as conventional physical therapy, treadmill, functional electrical stimulation, and robotic-assisted gait training. The velocity improvement, distance and functional score of walking as well as safety issues were comprehensively assessed in this study.

## Methods

Our systematic review and network meta-analysis were conducted in accordance with the Preferred Reporting Items for Systematic reviews and Meta-analyses (PRISMA) guidelines extension for network meta-analysis [18]. The study was registered at The International Prospective Register of Systematic Reviews; PROSPERO (ID: CRD42019145797).

One author identified all relevant studies in PubMed and Scopus databases as well as the research works published in academic journals and proceedings and previous systematic reviews with their reference lists up to October 2022. There was no language and status of publication restriction. Search terms comprised spinal cord injury, paralyzed, orthotic, physical therapy, electrical stimulation, treadmill, exoskeleton, robot, gait, velocity, walk and related terms as shown in Additional file 1: Table S1. Two reviewers selected RCTs based on titles and abstracts. If the decision could not be made, full articles were reviewed. Disagreements were resolved by discussion.

Eligible criteria were RCTs involving incomplete spinal cord injury patients with the American Spinal Cord Injury Association (ASIA) Impairment Scale classification grade B, C, and D [19] in comparison with any pairs of the following interventions: conventional physical therapy, functional electrical stimulation, treadmill, and robotic-assisted gait training. We excluded duplicated studies, those with insufficient data for pooling, and inaccessible full-text articles. The study factors were country of recruitment, sample sizes, study subject characteristics (age, time of injury, level of injury, ASIA impairment scale), and duration of interventions. The main outcome represented gait function including velocity (m/s), distance (m), WISCI [20], and WISCI II [21]. The secondary outcomes were any adverse events during gait training such as fall and pressure ulcer. Two reviewers independently retrieved the data using a standardized data extraction form. The quality assessment was separately evaluated by the two reviewers using the revised Cochrane risk-of-bias tool for randomized trials (RoB2) [22]. Each study was classified as having a low, high, or some concern of risk of bias. Any disagreements in data were resolved by team discussion.

### Statistical analysis

The direct meta-analysis was performed for each pair of the interventions when there were at least three studies. For continuous outcomes (velocity, distance, and WISCI score), unstandardized mean difference (USMD) with 95% confidence interval (CI) was estimated. The fixed-effects or random-effects model was used according to no or present heterogeneity (Cochrane's *Q* test *p* value < 0.1 or Higgins *I*^2^ > 25%), respectively. Sources of heterogeneity were explored by fitting each co-variable (level of injury, duration of injury, ASIA impairment scale, and time of training) in a meta-regression model. An asymmetric funnel plot or *p* value of the Egger’s test less than 0.05 indicated publication bias.

Network meta-analysis was indirectly compared with relative treatment effects across studies. We used linear regression to estimate mean differences for continuous outcomes. Multivariate random-effects meta-analysis with consistency model was performed to pool the relative treatment effects across the studies. Transitivity was indirectly explored by assessing the distribution of the effect of factors (age, gender, ASIA impairment scale, time of injury, and time of training) on the interested outcome between intervention arms. Consistency, agreement between direct and indirect comparisons, was evaluated by a global chi-square test using a design–treatment interaction inconsistency model. In case of significant global chi-square test (*p* < 0.05), a loop-specific approach was used to identify the treatment arms and studies that mainly contributed to the inconsistency. If inconsistency factors (IF) were greater than or equal to 2, patients’ characteristics (i.e., age, level of injury, duration of injury, lesion of injury, and ASIA impairment scale) among treatment arms of the closed loop were explored. A sensitivity analysis was performed by excluding the studies with different characteristics and rechecked the inconsistency assumption with a design-by-treatment interaction model. The probability of being the best intervention was assessed by the probability closest to 100. Surface under the cumulative ranking curve (SUCRA) and rankogram plot were used for ranking treatments [23]. The treatment effect in the future for each treatment regimen was estimated by the predictive interval. Publication bias was checked by using an adjusted funnel plot.

All analyses were performed using the STATA software package, version 16.0 (StataCorp, College Station, Texas, USA). The level of significance was < 0.05 for a two-sided *p* value and < 0.1 for a one-sided *p* value of heterogeneity test.

## Results

**Fig. 1:**
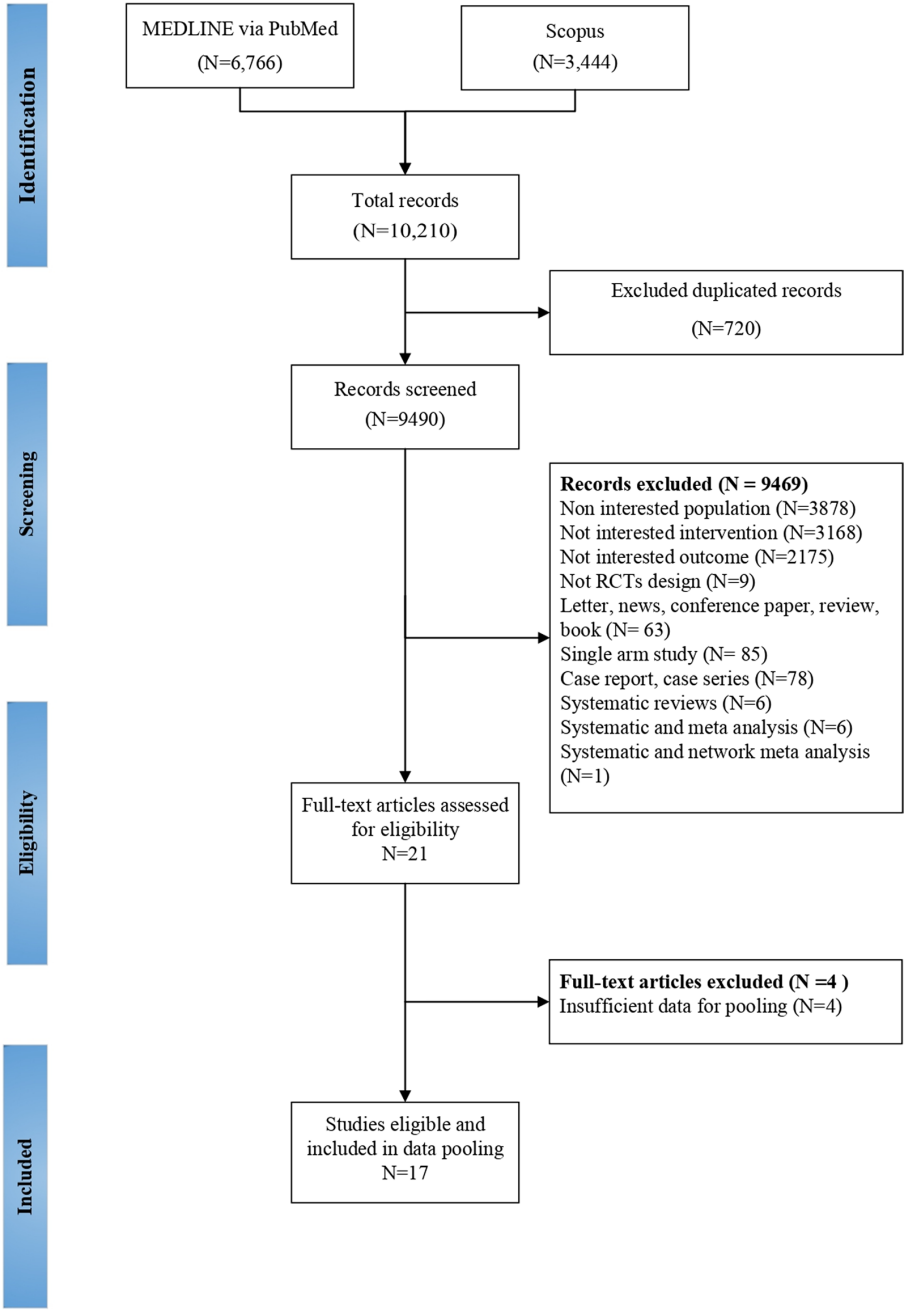
PRISMA flowchart

We identified 10,210 publications from PubMed (6766 studies) and Scopus (3444 studies) as shown in Additional file 1: Table S2. Of these, 720 duplicated studies were discarded. After screening titles, abstracts, and complete full-text review, 9469 studies were excluded leaving 17 RCTs (709 participants) for the review (Fig. 1). Fifteen studies were designed as parallel [17, 24–37], and the other two were crossover [38, 39]. Characteristics of included RCTs were the number of subjects ranging from 9 to 146, mean time of injury from 3 to 139 months, C1-L4 level, and ASIA impairment scale grade B-D (Table 1). They evaluated the effects of conventional physical therapy (94%) [24–39], robotic-assisted gait training (59%) [17, 25, 29–31, 33–35, 37, 38], treadmill (47%) [17, 24, 26, 28, 31, 32, 36, 39], functional electrical stimulation + treadmill (12%) [17, 27], and functional electrical stimulation (6%) [17]. Common duration of interventions across the included studies ranged from 3 to 16 weeks. Details of intervention and outcomes for the analysis are presented in Table 2.

**Table 1:** Characteristics of included studies

Author	Year	Country	RCT design	Conflict of interest	*n*	Age (years)	Male (%)	Time of injury (month)	Level of injury	ASIA scale
Hornby TG [31]	2005	USA	Parallel	No	35	N/A	N/A	N/A	Above T10	BCD
Dobkin B [32]	2006	USA	Parallel	No	146	26.8	76	N/A	C4-L3	BCD
Dobkin B [26]	2007	USA	Parallel	No	146	N/A	76	N/A	C4-L3	BCD
Alexeeva N [24]	2011	USA	Parallel	No	35	39.9	51	78.28	C2-T10	CD
Field-Fote EC [17]	2011	USA	Parallel	No	74	41.3	68	N/A	T10 or above	CD
Lucareli PR [28]	2011	Brazil	Parallel	No	30	31	46	9.85	C4-L2	CD
Alcobendas-Maestro M [29]	2012	Spain	Parallel	No	80	47.4	58	4.25	C2-T12	CD
Jaynie F [39]	2014	Canada	Crossover	No	22	46	N/A	64.2	C1-L1	N/A
Kapadia N [27]	2014	Canada	Parallel	No	34	55.3	61	114.36	C2-T12	CD
Labruyere R [38]	2014	Switzerland	Crossover	No	9	59	55	50	C4-T11	CD
Esclarin-Ruz A [33]	2014	Spain	Parallel	No	88	41.9	67	4.1	C1-L3	CD
Shin JC [35]	2014	Korea	Parallel	No	60	45.6	56	3.04	C-T	D
Tang Q [30]	2014	China	Parallel	No	30	38.6	100	N/A	T8-L3	D
Senthilvelkumar T [36]	2015	India	Parallel	No	16	35.2	68	5.9	C5-C8	C
Lin HD [34]	2016	China	Parallel	No	16	45.8	50	3.22	C4-L2	CD
Chang SH [25]	2018	USA	Parallel	No	9	57.7	55	138.84	C4-T12	CD
Edwards DJ [37]	2022	USA	Parallel	No	25	47	60	81.76	C1-L4	CD

*RCT*, randomized controlled trial, *ASIA*, the American Spinal Injury Association (B, sensory incomplete but no motor function is preserved below the level of injury, C, motor incomplete; muscle power grades 0-2, and D, motor incomplete; muscle power grade ≤ 3), *N/A*, not available

**Table 2:** Details of intervention and outcome assessments

Author	Year	Intervention	*n*	Duration of intervention (weeks)	Time measured after end of training	Outcome—measurement
Hornby TG [31]	2005	1. CPT: overground gait training	10	8	2 weeks after training	Distance—6 MWT
		2. TM: BWSTT (average speed 2 km/h)	10			
		3. RAGT: Lower exoskeleton (Lokomat) with BWS	10			
Dobkin B [32]	2006	1. CPT: overground gait training	18	12	Immediately after training	WISCI
		2. TM: BWSTT, stretching and gait training	27			
Dobkin B [26]	2007	1. CPT: overground gait training	37	12	Immediately after training	Velocity—15 MWT
		2. TM: BWSTT, stretching and gait training	34			Distance—6 MWT
Alexeeva N [24]	2011	1. CPT: overground gait training, balance, functional activity, stretching, strengthening and aerobic exercise	12	13	2 days after training	Velocity—10MWT
		2. TM: BWSTT (BWS 30%)	9			
Field-Fote EC [17]	2011	1. FES: FES on ankle dorsiflexor (common peroneal nerve), gait training (BWS 30%)	15	12	Immediately after training	Velocity—10MWT
		2. TM: unilateral or bilateral manual assistance for stepping BWS30 percent	17			Distance—2 MWT
		3. RAGT: lower exoskeleton, 100% guidance force to provide maximum assistance (BWS 30%)	14			
		4. FES + TM: FES on ankle dorsiflexor muscle (common peroneal nerve), BWSTT (BWS 30%)	18			
Lucareli PR [28]	2011	1. CPT: overground gait training	12	12	N/A	Velocity—Motion analysis
		2. TM: BWSTT (BWS 40%)	12			
						Distance—Motion analysis
Alcobendas-Maestro M [29]	2012	1. CPT: overground gait training	38	8	N/A	Velocity—10MWT
						Distance—6MWT
						WISCI
		2. RAGT: lower exoskeleton (Lokomat) (BWS 60%)	37			
Jaynie F [39]	2014	1. CPT: precision training	10	8	N/A	Velocity—10MWT
		2. TM: BWSTT	10			WISCI
Kapadia N [27]	2014	1. CPT: overground gait training, resistance and aerobic exercise	7	16	Immediately after training	Velocity—10MWT
		2. FES + TM: FES on quadriceps, hamstring, dorsiflexor, plantarflexor, BWSTT	14			Distance—6MWT
Labruyere R [38]	2014	1. CPT: strengthening exercise	9	4	Immediately after training	Velocity—10MWT
		2. RAGT: lower exoskeleton (average speed 1.5–2 km/h)	9			WISCI
Esclarin-Ruz A [33]	2014	1. CPT: overground gait training	36	8	N/A	Velocity—10MWT
		2. RAGT: lower exoskeleton (Lokomat) (BWS 60%)	36			Distance—6MWT
						WISCI
Shin JC [35]	2014	1. CPT: physical therapy (Bobath principle)	26	4	Within 2 days	WISCI
		2. RAGT: lower exoskeleton (Lokomat) (BWS 50%) (average speed fix 1.5 km/h)	27			
Tang Q [30]	2014	1. CPT: strengthening exercise (ergo bike load 60 W/ pedaling rate of 45 rpm)	15	N/A	N/A	Velocity—10MWT
		2. RAGT: lower exoskeleton 70% guidance force to provide assistance (Lokomat) (BWS 35%)(average speed 1.8 km\h)	15			
SenthilvelkumarT [36]	2015	1. CPT: overground gait training (BWS 40%)	8	8	N/A	WISCI
		2. TM: BWSTT	7			
Lin HD [34]	2016	1. CPT: overground gait training	8	8	N/A	WISCI
		2. RAGT: lower exoskeleton (A3)	8			
Chang SH [25]	2018	1. CPT: overground gait training, stretching, strengthening, balance, standing, sit to stand, stair	3	3	Immediately after training	Velocity—10MWT
		2. RAGT: lower exoskeleton (Ekso)	4			Distance—6MWT
Edwards DJ[37]	2022	1. CPT: overground gait training, mobility therapy	16	12	Immediately after training	Velocity—10MWT
		2. RAGT: lower exoskeleton (Ekso)	9			
						Distance—6MWT

*CPT*, conventional physical therapy, *FES*, functional electrical stimulation, *TM*, treadmill, *RAGT*, robotic-assisted gait training, *FES* + *TM*, functional electrical stimulation combined with treadmill, *BWS*, body weight support, *BWSTT*, body weight support treadmill training. *WISCI*, Walking Index for Spinal Cord Injury, *10MWT*, 10-min walk test, *6MWT*, 6-min walk test, *N/A*, not available

**Fig. 2:**
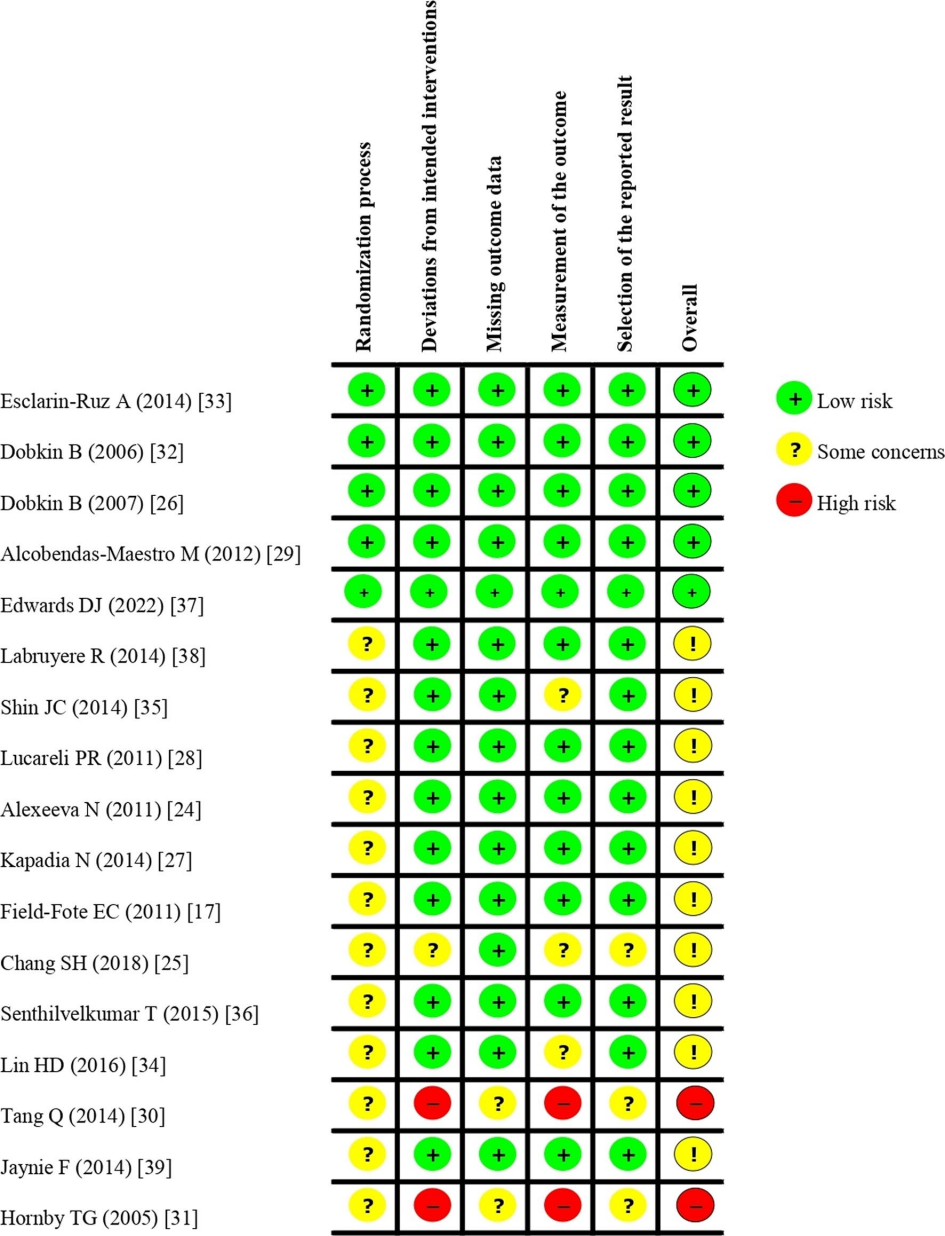
Risk of bias (RoB-2) of the included studies

The risk-of-bias assessment is shown in Fig. 2. The overall results were of medium quality (29% high [26, 29, 32, 33, 37], 59% moderate [17, 24, 25, 27, 28, 34–36, 38, 39], and 12% low quality [30, 31]). High-quality studies demonstrated overall low risk of biases. Moderate-quality studies had some concerns about randomization process and/or deviation from the intended intervention, measurement of the outcome, and selection of the reported results, while low-quality studies had a high risk of deviation from the intended intervention and measurement of the outcome.

### Direct meta-analysis

Direct meta-analysis involved 15 studies (601 patients) [24–26, 28–39] and three interventions (conventional physical therapy, treadmill, and robotic-assisted gait training). The studies reported the outcomes of velocity, distance, and WISCI without adverse events (Table 3). Publication biases are shown in Additional file 1: Fig. S1–S3.

**Table 3:** Pairwise meta-analysis

Included studies	Comparisons	Pairwise meta-analysis USMD (95% CI)
*Velocity (m/s)*		
4 studies [24, 26, 28, 39]	TM vs CPT	− 0.03 (− 0.14, 0.19)
6 studies [25, 29, 30, 33, 37, 38]	RAGT vs CPT	− 0.04 (− 0.04, 0.12)
*Distance (m)*		
3 studies [26, 28, 31]	TM vs CPT	75.87 (− 85.22, 236.96)
5 studies [25, 29, 31, 33, 37]	RAGT vs CPT	65.34 (− 36.26,166.92)
*WISCI*		
3 studies [32, 36, 39]	TM vs CPT	− 0.08 (− 0.93, 0.78)
5 studies [29, 33–35, 38]	RAGT vs CPT	3.28 (0.12, 6.45)

CPT, conventional physical therapy, FES, functional electrical stimulation, TM, treadmill, RAGT, robotic-assisted gait training, WISCI, Walking Index for Spinal Cord Injury, USMD, unstandardized mean difference, CI, confidence interval

### Velocity

Conventional physical therapy insignificantly increased velocity compared to treadmill [24, 26, 28, 39] (pooled USMD − 0.03 m/s, 95% CI − 0.14, 0.19; no heterogeneity *I*^2^ = 0%, *p* value = 0.69) and robotic-assisted gait training [25, 29, 30, 33, 37, 38] (pooled USMD 0.04 m/s, 95% CI − 0.04, 0.12; heterogeneity *I*^2^ = 70%, *p* value < 0.01). Publication bias of both pairs was absent (*p* value = 0.435, and 0.655, respectively). Subgroup analysis (Additional file 1: Fig. S4, S5) showed that robotic-assisted gait training improved velocity more than conventional physical therapy in acute-phase patients (time of injury < 6 months) (pooled USMD 0.1 m/s, 95% CI 0.05, 0.14; no heterogeneity *I*^2^ = 0%, *p* value = 0.76) and underwent at least 2-month duration of intervention (pooled USMD 0.1 m/s, 95% CI 0.06, 0.14; no heterogeneity *I*^2^ = 0%, *p* value = 0.91). Regarding to the level of cervical, thoracic, and lumbar spinal cord injury, subgroup analysis showed no significant difference of velocity between robotic-assisted gait training and conventional physical therapy (Additional file 1: Fig. S6).

### Distance

Conventional physical therapy was comparable with treadmill [26, 28, 31] (pooled USMD 76.00 m, 95% CI − 85.22, 236.96; heterogeneity *I*^2^ = 99%, *p* value < 0.01) and robotic-assisted gait training [25, 29, 31, 33, 37] (pooled USMD 65.34 m, 95% CI − 36.26, 166.92; heterogeneity *I*^2^ = 99%, *p* value < 0.001). There was no publication bias with *p* value = 0.930, and 0.075, respectively. Regarding subgroup analysis for acute-phase (Additional file 1: Fig. S7), robotic-assisted gait training provided longer walking distance than conventional physical therapy (pooled USMD 64.75 m, 95% CI 27.24, 102.27; heterogeneity *I*^2^ = 55%, *p* value = 0.14). Moreover, subgroup analysis for the level of cervical, thoracic, and lumbar spinal cord injury showed that robotic-assisted gait training improved distance more than conventional physical therapy (pooled USMD 40.45 m, 95% CI 14.69, 66.20; no heterogeneity *I*^2^ = 0%, *p* value = 0.89), Additional file 1: Fig. S8.

### WISCI

Compared to conventional physical therapy, robotic-assisted gait training significantly increased WISCI [29, 33–35, 38] (pooled USMD 3.28, 95% CI 0.12, 6.45; heterogeneity *I*^2^ = 90%, *p* value < 0.01), whereas treadmill showed no significant differences [32, 36, 39] (pooled USMD − 0.08, 95% CI − 0.93, 0.78; no heterogeneity *I*^2^ = 0%, *p* value = 0.73). Publication bias of both pairs was absent (*p* value = 0.160, and 0.727, respectively). Regarding subgroup analysis for studies included cervical, thoracic, and lumbar spinal cord injury, robotic-assisted gait training improved WISCI score more than conventional physical therapy (pooled USMD 2.86, 95% CI 0.07, 5.66; heterogeneity *I*^2^ = 28.09%, *p* value = 0.24), Additional file 1: Fig. S9.

### Network meta-analysis

**Fig. 3:**
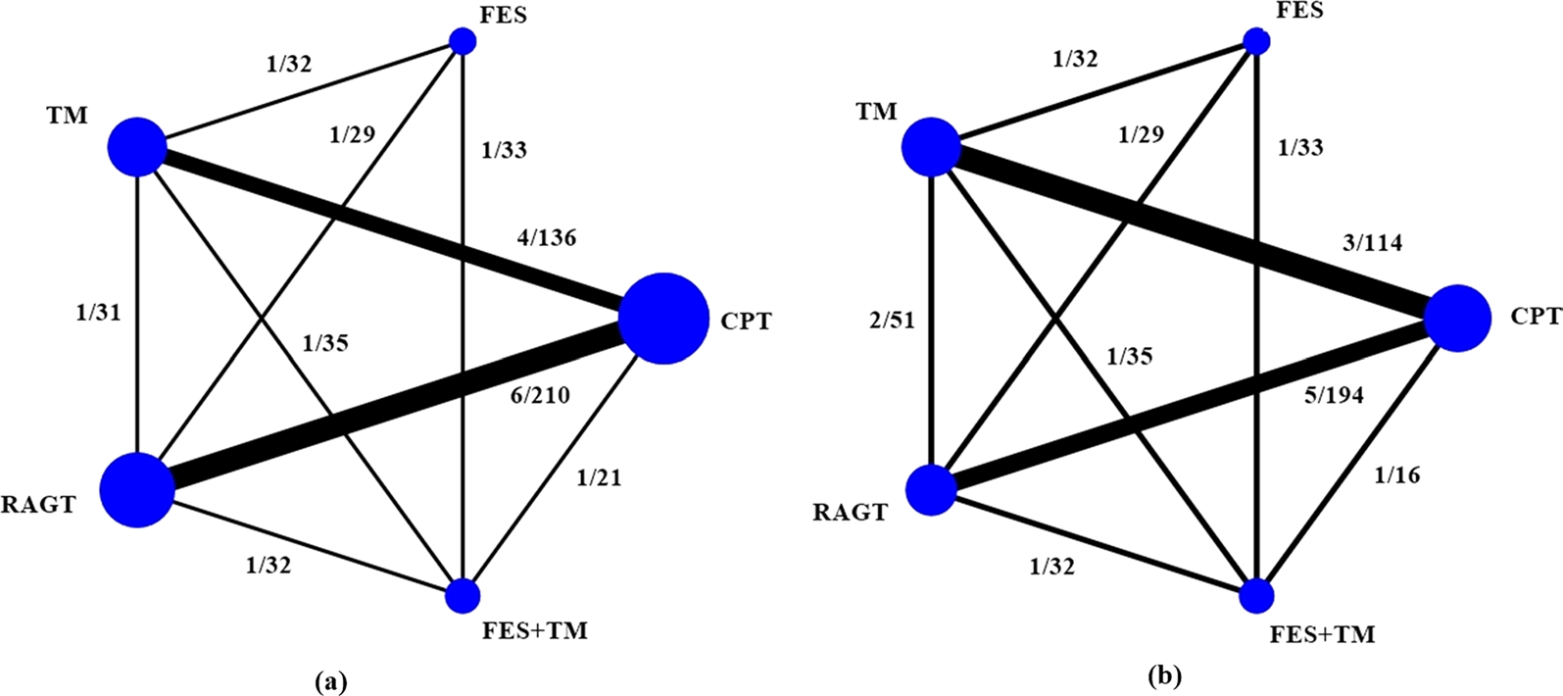
Network of possible comparisons between intervention and comparator for **a** velocity and **b** distance. CPT = conventional physical therapy, FES = functional electrical stimulation, TM = treadmill, RAGT = robotic-assisted gait training, FES + TM = functional electrical stimulation combined with treadmill. The width of the lines is proportional to the numbers of studies and the size of the nodes is proportional to the sample size. Numbers along the lines refer to numbers of studies/numbers of patients corresponding to direct comparisons

Network meta-analysis included 13 studies (709 patients) [17, 24–31, 33, 37–39], 5 interventions (conventional physical therapy, functional electrical stimulation, treadmill, robotic-assisted gait training, functional electrical stimulation + treadmill), indirect and 9 direct comparisons (Fig. 3). Only velocity and distance outcomes had adequate studies to be pooled (Table 4).

**Table 4:** Multiple treatment comparison of the network for velocity and distance outcome

Reference treatment	Mean difference				
	CPT	FES + TM	RAGT	TM	FES
*Velocity, m/s*					
CPT	**17.8, 0.8**	0.05 (− 0.08,0.18)	0.04 (− 0.03,0.10)	0.05 (− 0.07,0.17)	0.12 (− 0.07,0.31)
FES + TM	− 0.05 (− 0.18,0.08)	**48.6,11.6**	− 0.01 (− 0.14,0.11)	0.00 (− 0.14,0.14)	0.07 (− 0.12,0.26)
RAGT	− 0.04 (− 0.10,0.03)	0.01 (− 0.11,0.14)	**47.0,6.7**	0.02 (− 0.10,0.13)	0.08 (− 0.10,0.26)
TM	− 0.05 (− 0.17,0.07)	− 0.00 (− 0.14,0.14)	− 0.02 (− 0.13,0.10)	**54.4,14.3**	0.07 (− 0.12,0.25)
FES	− 0.12 (− 0.31,0.07)	− 0.07 (− 0.26,0.12)	− 0.08 (− 0.26,0.10)	− 0.07 (− 0.25,0.12)	**82.1,66.6**
*Distance, m*					
CPT	**10.8, 0.1**	68.64 (− 40.39, 177.67)	37.58 (− 32.58, 107.73)	73.36 (− 2.78, 149.50)	76.26 (− 59.68, 212.20)
FES + TM	− 68.64 (− 177.67, 40.39)	**60.6, 23.7**	− 31.06 (− 144.60, 82.47)	4.72 (− 110.18, 119.62)	7.62 (− 132.66, 147.91)
RAGT	− 37.58 (− 107.73, 32.58)	31.60 (− 82.47, 144.60)	**41.9, 6.7**	35.78 (− 56.05, 127.61)	38.69 (− 95.66, 173.03)
TM	− 73.36 (− 149.50, 2.78)	− 4.72 (− 119.62, 110.18)	− 35.78 (− 127.61, 56.05)	**69.4, 29.8**	2.90 (− 131.89, 137.70)
FES	− 76.26 (− 212.20, 59.68)	− 7.62 (− 147.91, 132.66)	− 38.69 (− 173.03, 95.66)	− 2.90 (− 137.70, 131.89)	**67.4, 39.7**

*CPT*, conventional physical therapy, *FES*, functional electrical stimulation, *TM*, treadmill, *RAGT*, robotic-assisted gait training, *FES* + *TM*, functional electrical stimulation combined with treadmill, bold letters determine surface under the cumulative ranking curve (SUCRA), and probability of being the best treatment, respectively

### Velocity

**Fig. 4:**
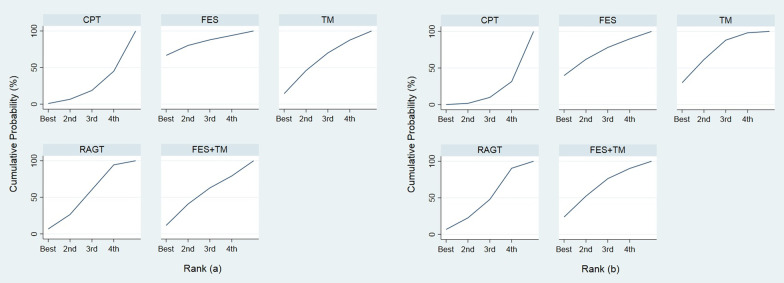
Rankograms for **a** velocity and **b** distance network showing the probability for each intervention being the best to improve walking velocity in patients with incomplete spinal cord injury, CPT = conventional physical therapy, FES = functional electrical stimulation, TM = treadmill, RAGT = robotic-assisted gait training, FES + TM = functional electrical stimulation combined with treadmill

Twelve studies (10 RCTs [17, 24–30, 33, 37] and 2 crossover design [38, 39]) involving 559 patients and the velocity outcome were pooled. There was no evidence of inconsistency (global chi-square = 0.90, *p* value = 0.64). The network map was constructed for multiple comparisons of 5 interventions and also 9 direct comparisons (Fig. 3). Functional electrical stimulation showed insignificant treatment benefit when compared to conventional physical therapy, treadmill, robotic-assisted gait training, and functional electrical stimulation + treadmill with pooled USMD (95% CI) of 0.12 (− 0.07, 0.31), 0.07 (− 0.12, 0.25), 0.08 (− 0.10, 0.26), and 0.07 (− 0.12, 0.26) m/s, respectively (Table 4). From Fig. 4, functional electrical stimulation had the highest probability of being the best velocity improvement (66.6%), followed by treadmill (14.3%), functional electrical stimulation + treadmill (11.6%), robotic-assisted gait training (6.7%), and conventional physical therapy (0.8%).

### Distance

Data from 9 RCTs [17, 25–29, 31, 33, 37] including 496 patients and distance outcomes were analyzed. The design-by-treatment interaction model showed evidence of inconsistency (global chi-square = 60.50, df = 8, *p* value < 0.001). Sensitivity analysis was performed by excluding one study comparing robotic-assisted gait training and conventional therapy [31] since the authors did not report the number of participants in each ASIA impairment scale. The model inconsistency was reduced (global chi-square = 0.26, *p* value = 0.880). The network map was constructed for multiple comparisons of 5 interventions and 9 direct comparisons (Fig. 3). Treatment effect of functional electrical stimulation was superior to conventional physical therapy, treadmill, robotic-assisted gait training and functional electrical stimulation + treadmill with pooled USMD (95% CI) of 76.26 (− 59.68, 212.20), 2.90 (− 131.89, 137.70), 38.69 (− 95.66, 173.03), and 7.62 (− 132.66, 147.91) m, respectively (Table 4). Functional electrical stimulation was the highest probability of being the best intervention (39.7), followed by treadmill (29.8), functional electrical stimulation + treadmill (23.7), robotic-assisted gait training (6.7), and conventional physical therapy (0.1) (Fig. 4).

Nonsignificant predictive interval plots of velocity and distance outcomes indicated that future studies tend to produce similar results to ours (Additional file 1: Fig. S10, S11).

## Discussion

Our systematic review and network meta-analysis evaluated the treatment effects among 5 gait training interventions to improve walking ability of incomplete spinal cord injury patients. Most of the included 17 RCTs were low to some of concerned risks of biases. From direct meta-analysis, robotic-assisted gait training tended to provide faster walking rate, longer distance, and significantly higher WISCI scores than conventional physical therapy. With regards to the network meta-analysis, there was no significant differences of velocity and distance between gait training interventions. Functional electrical stimulation tended to be the most effective gait training method for both velocity and distance outcomes (probability of being the best treatment 66.6% and 39.7%, respectively) followed by treadmill, functional electrical stimulation + treadmill, robotic-assisted gait training, and conventional physical therapy, respectively.

Robotic-assisted gait training provided better functional level than conventional physical therapy with nonsignificant different speed and distance [13]. Our reviews demonstrated significant 3.3 WISCI score improvement, and subgroup analyses among acute-phase patients with robotic-assisted gait training for at least 2 months significantly increased 0.1 m/s walking rate, and 64.75 m longer distance than the conventional physical therapy. Moreover, subgroup analyses of the level of cervical, thoracic, and lumbar spinal cord injury with robotic-assisted gait training significantly increased 40.45 m longer distance and 2.86 WISCI score than the conventional physical therapy. This might be due to low muscle tone during acute stage. Spasticity usually started 2–6 months after spinal cord injury [40–42]. Joint stiffness, muscle shortening [43], and neural plasticity [44, 45] would impact functional independence and gait training program [43, 46]. The higher the level of spinal cord injury, the more dysfunction of the body, and gait improvement. Either assistant or resistant robotic-assisted gait training could offer advantages in limb control, muscle strengths [47], physiological and reproducible gait patterns [48]. A network meta-analysis by Ma et al. [11] demonstrated that robotic-assisted gait training with overground training improved WISCI score with the highest SUCRA value (88.5) when compared to body weight support treadmill training and body-weight-supported overground training. Additional physical therapy, time of training, and velocity measurement might influence the effects of treatment. However, our subgroup analysis did not find significant difference.

According to our network meta-analysis, functional electrical stimulation was preferred to functional electrical stimulation + treadmill and treadmill in terms of velocity and distance without statistically significance. Functional electrical stimulation can enhance gait, muscle strength, and cardiorespiratory fitness for spinal cord injury patients [49]. Overground training might allow lifting, assisting, and walking variability [50, 51]. Field-Fote et al. [17] reported that functional electrical stimulation combined with overground training facilitated walking tasks greater than its combination with body-weight-supported treadmill training. However, including highly impaired participants in functional electrical stimulation + treadmill may cause inferior results [17]. Treadmill alone dominated the effect of walking velocity and distance compared to functional electrical stimulation + treadmill. This method triggers central pattern generators (CPGs) within the spinal cord [52] and improves stride and balance [53], whereas functional electrical stimulation improves foot drop and coordination of the lower limb movement [54–56]. Previous research works showed that combined functional electrical stimulation + treadmill could improve velocity and distance more than treadmill [17, 54]. Nevertheless, Kesar et al. [54] found nonsignificant difference of percent propulsion of ankle ground reaction force which correlated with velocity [57, 58]. This controversy still needs more evidence to support results. Robotic-assisted gait training and conventional physical therapy showed lower velocity and distance improvement than functional electrical stimulation. Even though these three gait rehabilitations build up muscle strengths and balance in spinal cord injury patients [11–13], functional electrical stimulation specifically activates weak muscles to improve foot clearance, stride length [59] as well as walking velocity and distance. Moreover, functional electrical stimulation can alleviate pain and spasticity which might reinforce gait training [60].

Strengths of our study are rigorous methodology as research questions focusing on incomplete spinal cord injury, following PRISMA guidelines, and including recently published RCTs without language restriction. Our search terms covered common gait training interventions and assessed specific outcomes as walking velocity, distance, and WISCI score. We investigated sources of heterogeneity by meta-regression and subgroup analysis and conducted sensitivity analysis by removing the heterogonous studies. Limitations of this review are small number of included studies in some comparison arms leading to imprecise/insignificant estimated treatment effects. Although 88% of included RCTs had low to some concern risk of bias, some of them did not report concealment (selection bias) and were unable to blind outcome assessors (measurement bias). Moreover, weighting methods for risk of biases did not apply for the analysis. The treatment effect of each intervention was based on average 2-month training which is inapplicable for long-term results. No included studies reported adverse events (fall, fracture, rash, and pressure ulcers) during the training period. We, therefore, are unable to presume gait training safety for incomplete spinal cord injury.

Robotic-assisted gait training significantly improves WISCI score more than conventional physical therapy. Velocity and distance of walking appear to be significant in acute phase of incomplete spinal cord injury patients. However, this gait training is very expensive (40,000–150,000 USD) [61] and requires cheaper price manufacturing with adapted home use. Among the 5 gait trainings, functional electrical stimulation tended to be the most effective intervention to improve velocity and distance of walking. Since functional electrical stimulation had low number of treatment arms and was not included in the direct meta-analysis, only indirect comparisons have been made in the network meta-analysis with nonsignificant difference from other interventions. The interpretation of the final results should be with caution. However, this modality is practical and cost-effective [62, 63] and provides good outcomes with affordable price. Further RCTs are recommended to compare robotic-assisted gait training and functional electrical stimulation to achieve a proper conclusion.

## Supplementary Information

**Additional file 1. Table S1.** Search strategy in Medline via PubMed. **Table S2.** Search strategy in Scopus. **Fig. S1.** Funnel plots of (**a**) treadmill (TM) versus conventional physical therapy (CPT) (**b**) robotic assisted gait training (RAGT) versus conventional physical therapy and (**c**) contour funnel plot of plot robotic assisted gait training versus conventional physical therapy. **Fig. S2.** Funnel plots of (**a**) treadmill (TM) versus convention physical therapy (CPT) (**b**) robotic assisted gait training (RAGT) versus conventional physical therapy, and contour funnel plot of (**c**) treadmill versus convention physical therapy and (**d**) robotic assisted gait training versus conventional physical therapy for distance, **Fig. S3.** Funnel plots of Walking Index Spinal Cord injury (WISCI) outcome, (**a**) treadmill versus conventional physical therapy (**b**) robotic assisted gait training versus conventional physical therapy and contour funnel plot and (**c**) robotic assisted gait training versus conventional physical therapy. **Fig. S4.** Subgroup analysis of the time of injury between robotic assisted gait training versus conventional physical therapy on velocity of walking (m/s) in patients with incomplete spinal cord injury, **Fig. S5.** Subgroup analysis of time of training between robotic assist gait training versus conventional physical therapy on velocity of walking (m/s) in patients with incomplete spinal cord injury. **Fig. S6.** Subgroup analysis of the level of spinal cord injury between robotic assisted gait training versus conventional physical therapy on velocity of walking (m/s) in patients with incomplete spinal cord injury. **Fig. S7.** Subgroup analysis of time of injury between robotic assisted gait training versus conventional physical therapy on distance of walking (m) in patients with incomplete spinal cord injury.**Fig. S8.** Subgroup analysis of the level of spinal cord injury between robotic assisted gait training versus conventional physical therapy on distance of walking (m) in patients with incomplete spinal cord injury. **Fig. S9.** Subgroup analysis of the level of spinal cord injury between robotic assisted gait training versus conventional physical therapy on Walking Index Spinal Cord injury (WISCI) outcome in patients with incomplete spinal cord injury. **Fig. S10.** Predictive interval plots of mean difference for velocity (m/s) of 10 pairwise comparisons estimated; values in right column showed mean difference (95% confidence interval) (95% predictive interval). **Fig. S11.** Predictive interval plots of mean difference for distance (m) of 10 pairwise comparisons estimated; Values in right column showed mean difference (95% confidence interval) (95% predictive interval).

## Data Availability

The datasets used and/or analyzed during the current study are available from the corresponding author upon reasonable request.

## Abbreviations

WISCI: Walking Index Spinal Cord Injury
RCT: Randomized controlled trial
NMA: Network meta-analysis
USMD: Unstandardized mean difference
CI: Confidence interval
SUCRA: Surface under the cumulative ranking curve
PRISMA: Preferred reporting items for systematic reviews and meta-analyses
ASIA: With the American spinal cord injury association
RoB-2: The revised Cochrane risk-of-bias tool for randomized trials
