# Supplementary material for: The efficacy of gait rehabilitations for the treatment of incomplete spinal cord injury: a systematic review and network meta-analysis
Source: J Orthop Surg Res. 2023 Jan 23;18:60. doi: 10.1186/s13018-022-03459-w (PMC9869518; doi:10.1186/s13018-022-03459-w)
Supplement: Supplementary file 1 — Additional file 1. Table S1. Search strategy in Medline via PubMed. Table S2. Search strategy in Scopus. Fig. S1. Funnel plots of (a) treadmill (TM) versus conventional physical therapy (CPT) (b) robotic assisted gait training (RAGT) versus conventional physical therapy and (c) contour funnel plot of plot robotic assisted gait training versus conventional physical therapy. Fig. S2. Funnel plots of (a) treadmill (TM) versus convention physical therapy (CPT) (b) robotic assisted gait training (RAGT) versus conventional physical therapy, and contour funnel plot of (c) treadmill versus convention physical therapy and (d) robotic assisted gait training versus conventional physical therapy for distance, Fig. S3. Funnel plots of Walking Index Spinal Cord injury (WISCI) outcome, (a) treadmill versus conventional physical therapy (b) robotic assisted gait training versus conventional physical therapy and contour funnel plot and (c) robotic assisted gait training versus conventional physical therapy. Fig. S4. Subgroup analysis of the time of injury between robotic assisted gait training versus conventional physical therapy on velocity of walking (m/s) in patients with incomplete spinal cord injury, Fig. S5. Subgroup analysis of time of training between robotic assist gait training versus conventional physical therapy on velocity of walking (m/s) in patients with incomplete spinal cord injury. Fig. S6. Subgroup analysis of the level of spinal cord injury between robotic assisted gait training versus conventional physical therapy on velocity of walking (m/s) in patients with incomplete spinal cord injury. Fig. S7. Subgroup analysis of time of injury between robotic assisted gait training versus conventional physical therapy on distance of walking (m) in patients with incomplete spinal cord injury.Fig. S8. Subgroup analysis of the level of spinal cord injury between robotic assisted gait training versus conventional physical therapy on distance of walking (m) in patients with in [file 13018_2022_3459_MOESM1_ESM.docx]

**Supplementary**

**Table S1** Search strategy in Medline via PubMed

| **Domain** | **Search number** | **Query** | **Items Found** |
| --- | --- | --- | --- |
| **Patient (P)** | | | |
| **P1** | #1 | Search "spinal cord inju*" | 76,628 |
|  | #2 | Search "spinal cord injuries"[MeSH Terms] | 54,282 |
|  | #3 | Search "spinal cord trauma" | 946 |
|  | #4 | Search #1 OR #1 OR #3 | 86,503 |
| **P2** | #5 | Search Paralysis | 120,675 |
|  | #6 | Search Paralyzed | 8,708 |
|  | #7 | Search Paraly* | 181,681 |
|  | #8 | Search #5 OR #6 OR #7 | 133,920 |
|  | #9 | Search #4 OR #8 | 209,396 |
| **Domain** | **Search number** | **Query** | **Items Found** |
| **Intervention(I)** | | | |
| **Orthotics** | #10 | Search orthotics | 19,233 |
|  | #11 | Search orthotic | 19,233 |
|  | #12 | Search orthosis | 18,637 |
|  | #13 | Search orthoses | 18,069 |
|  | #14 | Search Exoskeleton | 4,428 |
|  | #15 | Search Robot* | 83,417 |
|  | #16 | Search Hybrid* | 526,846 |
| **Treadmill** | #17 | Search "treadmill" | 35,682 |
| **FES** | #18 | Search "* electrical stimulation" | 195,091 |
| **CPT** | #19 | Search "physical therap*" | 107,757 |
|  | #20 | Search "Physical therapy" | 102,565 |
|  | #21 | Search "physical therapist" | 3,648 |
|  | #22 | Search "Physiotherap*" | 69,392 |
|  | #23 | Search "Physiotherapy" | 59,853 |
|  | #24 | Search "Physiotherapist" | 4,877 |
|  | #25 | Search "Mobilization" | 60,867 |
|  | #26 | Search gait* | 95,440 |
|  | #27 | Search walk* | 230,992 |
|  | #28 | Search exercise | 1,724,001 |
|  | #29 | Search #10 OR #11 OR #12 OR #13 OR #14 OR #15 OR #16 OR #17 OR #18 OR #19 OR #20 OR #21 OR #22 OR #23 OR #24 OR #25 OR #26 OR #27 OR #28 OR #29 |  |

**Table S1** Search strategy in Medline via PubMed (cont.)

| **Domain** | **Search number** | **Query** | **Items Found** |
| --- | --- | --- | --- |
| **Outcome(O)** | #30 | Search Velocity | 246,252 |
|  | #31 | Search Speed | 223,036 |
|  | #32 | Search Distance | 337,669 |
|  | #33 | Search *walk* | 230,992 |
|  | #34 | Search "The Walking Index" | 15,945 |
|  | #35 | Search #30 OR #31 OR #32 OR #33 OR #34 OR #35 | 960,320 |
| **PIO** | #36 | Search #9 AND #29 AND #35 | 6,766 |

**Table S2** Search strategy in Scopus

| **Domain** | **Search number** | **Query** | | **Items Found** |
| --- | --- | --- | --- | --- |
| **Patient(P)** | | | | |
| **P1** | #1 | Search “spinal cord injury” | 227,426 | |
|  | #2 | Search “spinal cord trauma” | 9,235 | |
|  | #3 | Search "spinal cord injury" OR"spinal cord trauma" | 229,514 | |
|  | #4 | Search Paraplegia | 89,106 | |
| **P2** | #5 | Search Paralyze | 8,710 | |
|  | #6 | Search paraplegia OR paralyze | 97,538 | |
|  | #7 | Search #3 AND #6 | 35,357 | |
| **Domain** | **Search number** | **Query** | **Items Found** | |
| **Intervention(I)** | #8 | Search orthotics | 31,394 | |
| **Orthotics** | #9 | Search orthosis | 68,366 | |
|  | #10 | Search orthoses | 68,366 | |
|  | #11 | Search Exoskeleton | 42,246 | |
|  | #12 | Search "Robot assisted gait" | 2,960 | |
|  | #13 | Search "Robotic assisted gait" | 1,027 | |
|  | #14 | Search "Hybrid assistive limb" | 1,118 | |
| **Treadmill** | #15 | Search treadmill | 132,994 | |
| **FES** | #16 | Search "functional electrical stimulation" | 18,129 | |
| **CPT** | #17 | Search “physical therapy” | 354,595 | |
|  | #18 | Search "Physiotherapy" | 255,649 | |
|  | #19 | Search "Strengthening exercise " | 8,086 | |
|  | #20 | Search "Aerobic exercise" | 90,094 | |
|  | #21 | Search "Stretching exercise" | 8,894 | |
|  | #22 | Search gait | 337,235 | |
|  | #23 | Search walk | 471,465 | |
|  | #24 | Search #8 OR #9 OR #10 OR #11 OR #12 OR #13 OR #14 OR #15 OR #16 OR #17 OR #18 OR #19 OR #20 OR #21 OR #22 OR #23 | 1,376,348 | |

**Table S2** Search strategy in Scopus (cont.)

| **Domain** | **Search number** | **Query** | **Items Found** |
| --- | --- | --- | --- |
| **Outcome(O)** | #25 | Search Velocity | 2,448,977 |
|  | #26 | Search Speed | 3,282,446 |
|  | #27 | Search Distance | 2,908,789 |
|  | #28 | Search "10 minute walk test " | 25 |
|  | #29 | Search "6 minute walk test " | 11,381 |
|  | #30 | Search "ten minute walk test " | 69 |
|  | #31 | Search "six minute walk test " | 23,543 |
|  | #32 | Search "Walking Index" | 712 |
|  | #33 | Search #25 OR #26 OR #27 OR #28 OR #29 OR #30 OR #31 OR #32 | 7,784,679 |
| **PIO** | #34 | Search #7 AND #24 AND #33 | 3,444 |


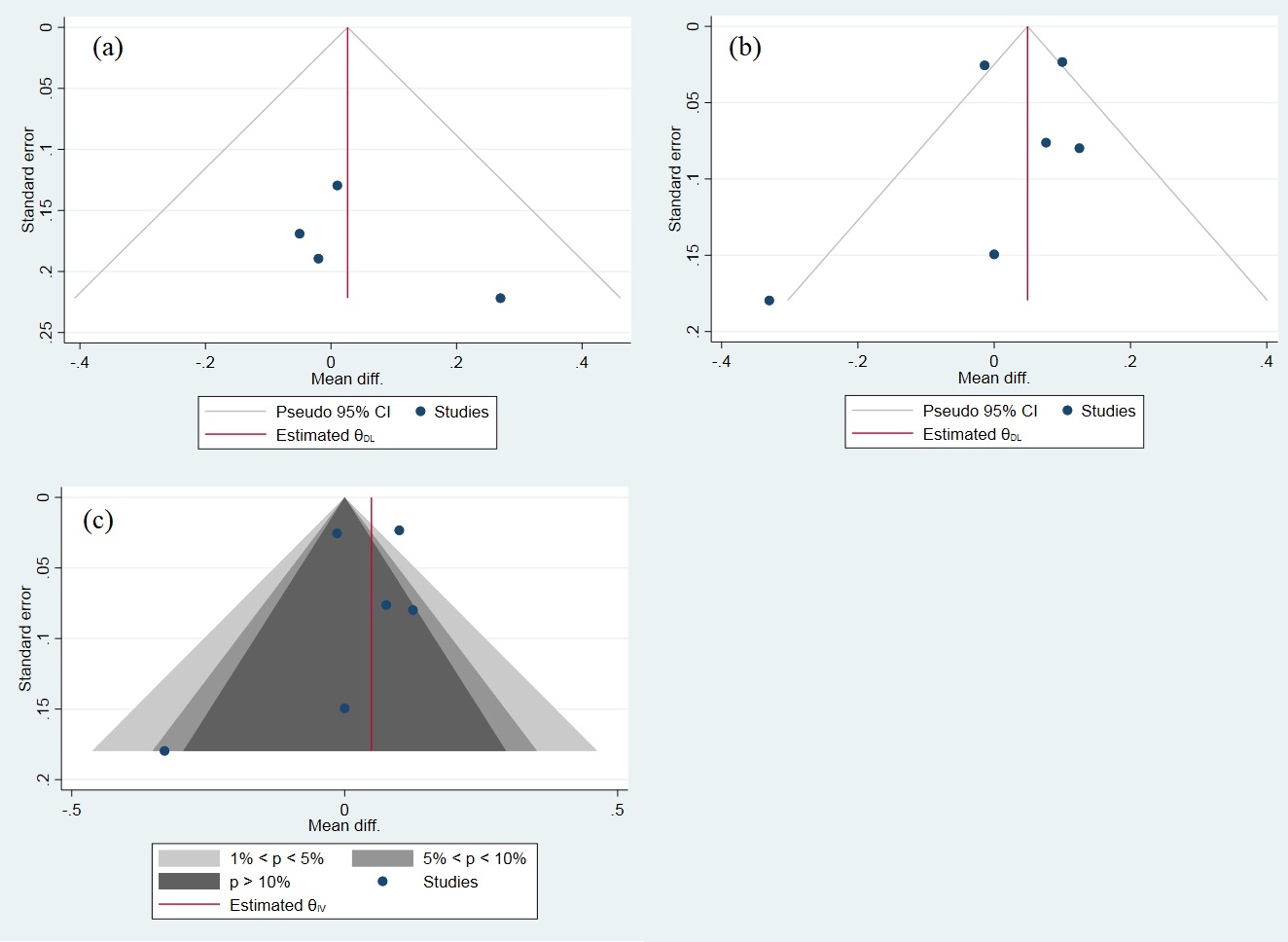


**Fig. S1** Funnel plots of (a) treadmill (TM) versus conventional physical therapy (CPT) (b) robotic assisted gait training (RAGT) versus conventional physical therapy and (c) contour funnel plot of plot robotic assisted gait training versus conventional physical therapy


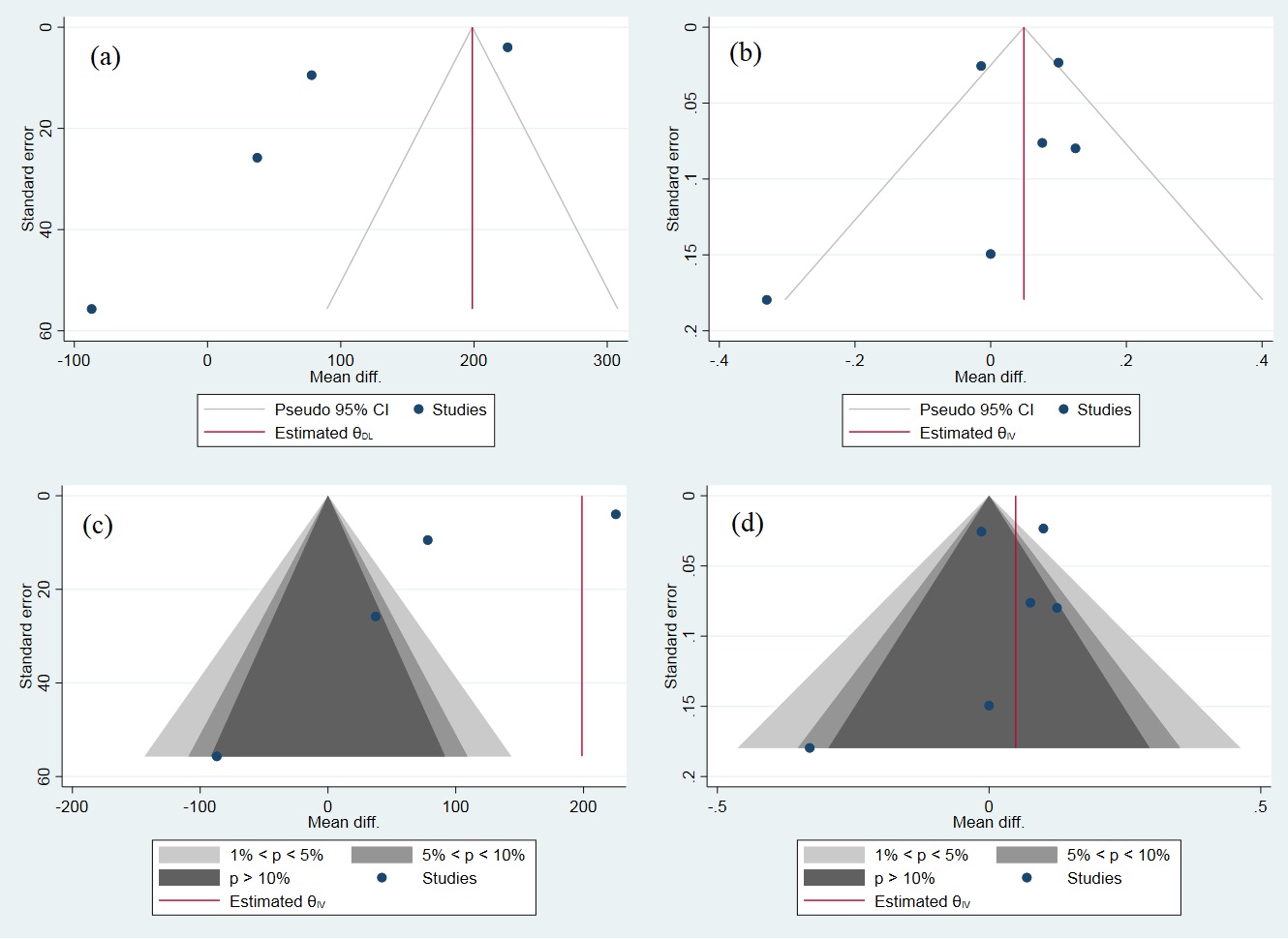


**Fig. S2** Funnel plots of (a) treadmill (TM) versus convention physical therapy (CPT) (b) robotic assisted gait training (RAGT) versus conventional physical therapy, and contour funnel plot of (c) treadmill versus convention physical therapy and (d) robotic assisted gait training versus conventional physical therapy for distance


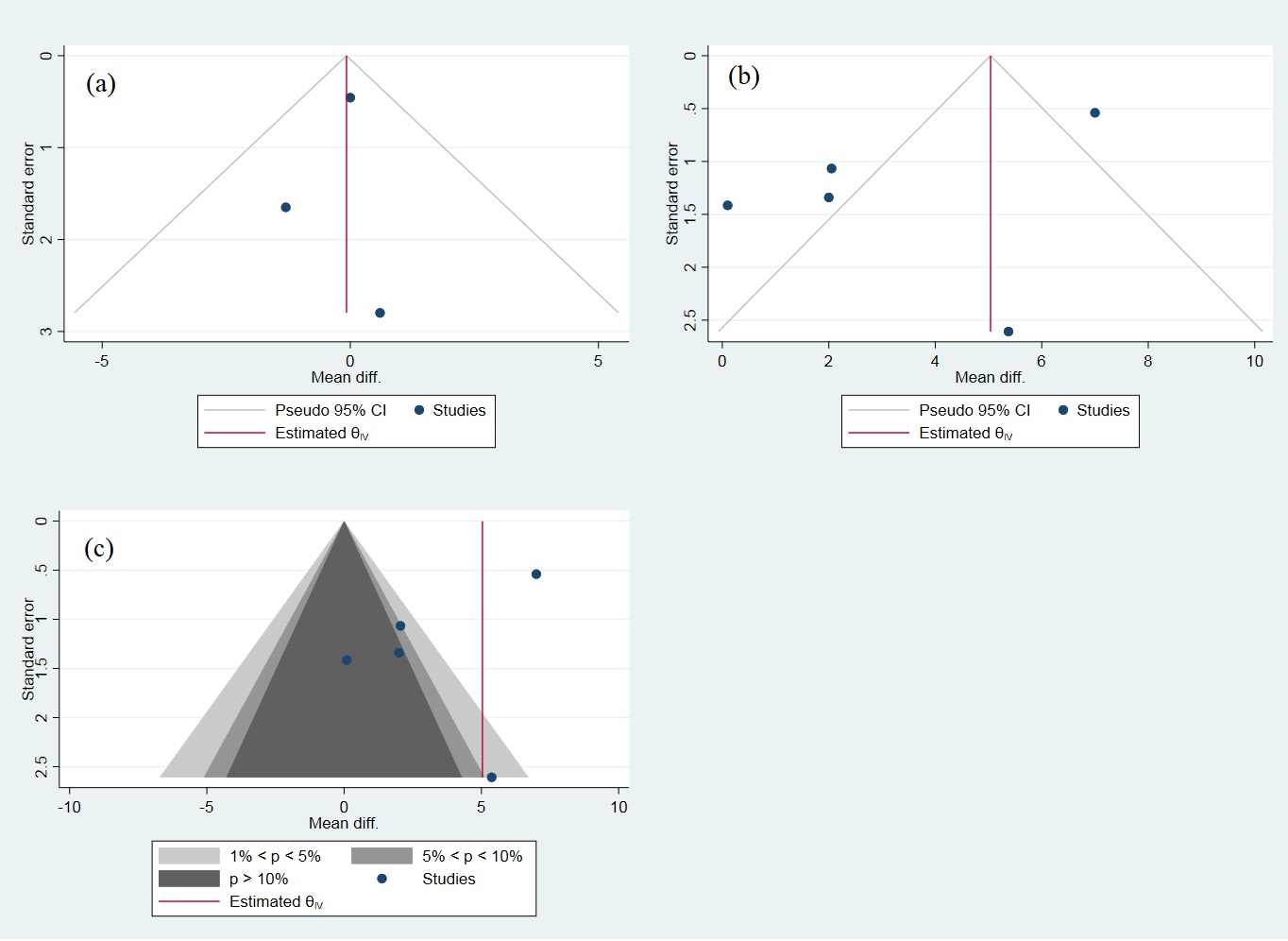


**Fig. S3** Funnel plots of Walking Index Spinal Cord injury (WISCI) outcome, (a) treadmill versus conventional physical therapy (b) robotic assisted gait training versus conventional physical therapy and contour funnel plot and (c) robotic assisted gait training versus conventional physical therapy

**
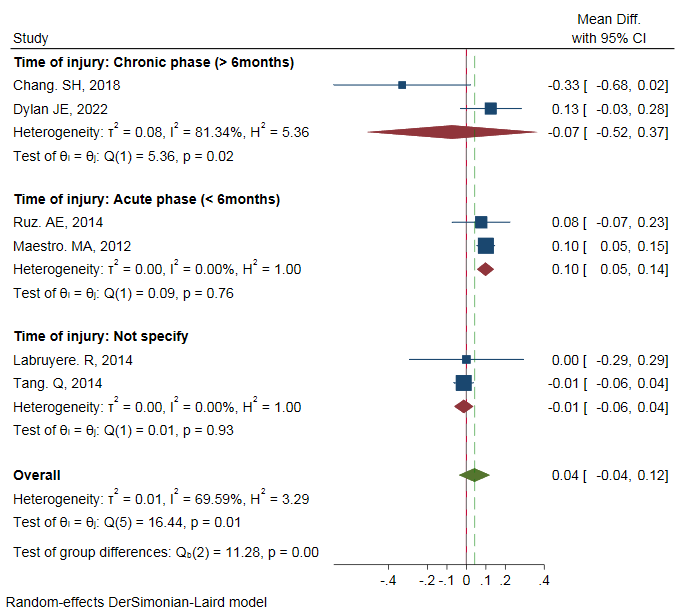
**

**Fig. S4** Subgroup analysis of the time of injury between robotic assisted gait training versus conventional physical therapy on velocity of walking (m/s) in patients with incomplete spinal cord injury

**
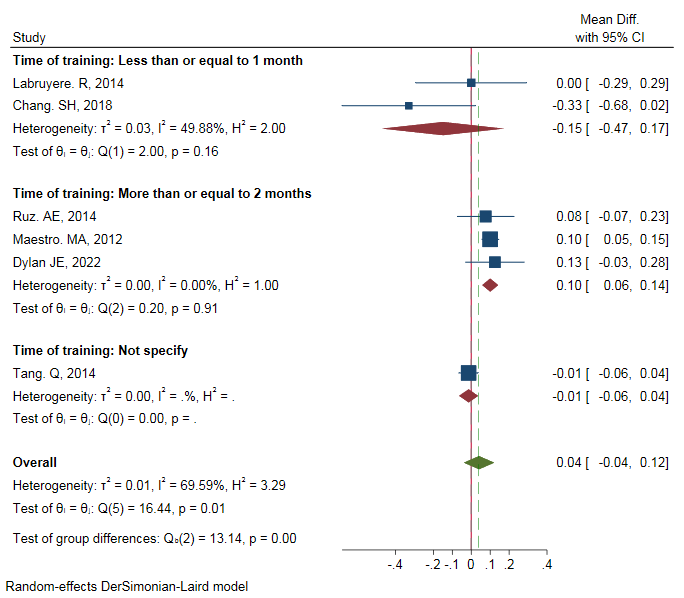
**

**Fig. S5** Subgroup analysis of time of training between robotic assist gait training versus conventional physical therapy on velocity of walking (m/s) in patients with incomplete spinal cord injury

**
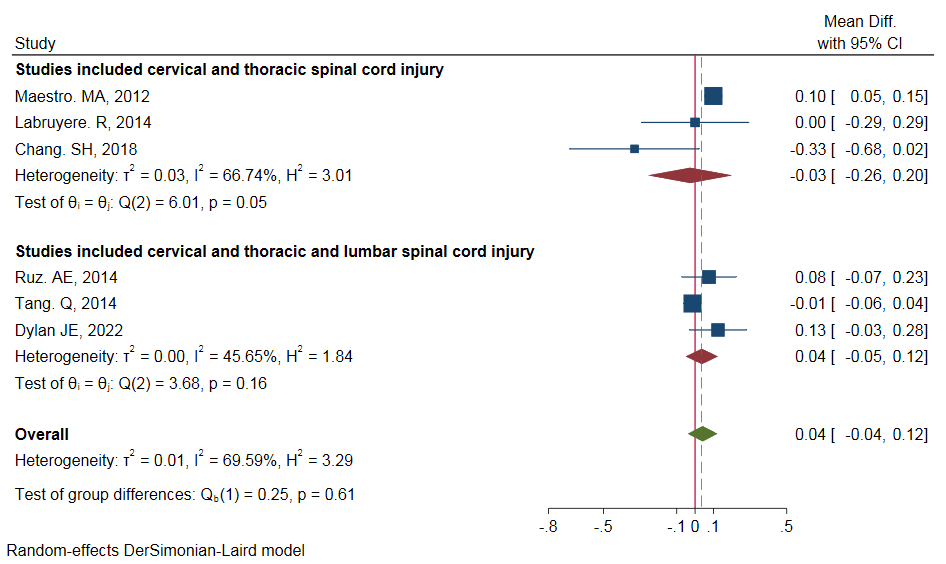
**

**Fig. S6** Subgroup analysis of the level of spinal cord injury between robotic assisted gait training versus conventional physical therapy on velocity of walking (m/s) in patients with incomplete spinal cord injury

**
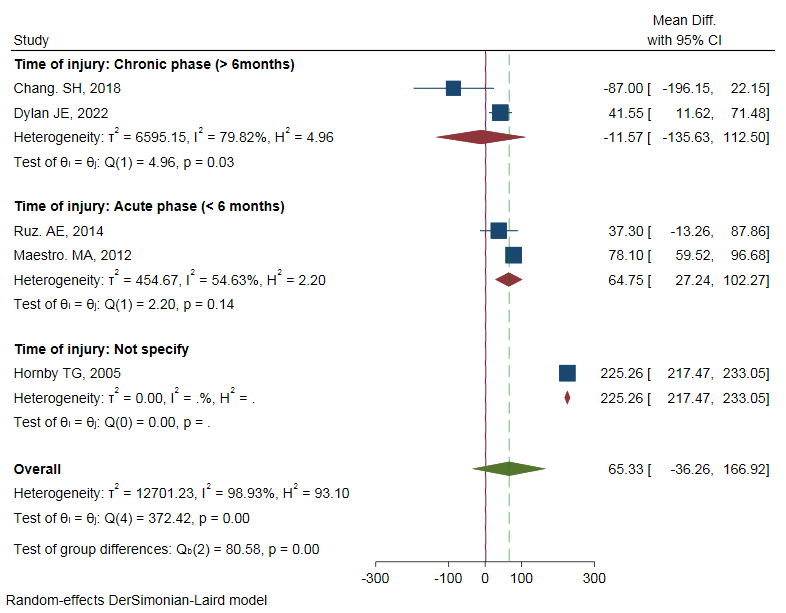
**

**Fig. S7** Subgroup analysis of time of injury between robotic assisted gait training versus conventional physical therapy on distance of walking (m) in patients with incomplete spinal cord injury


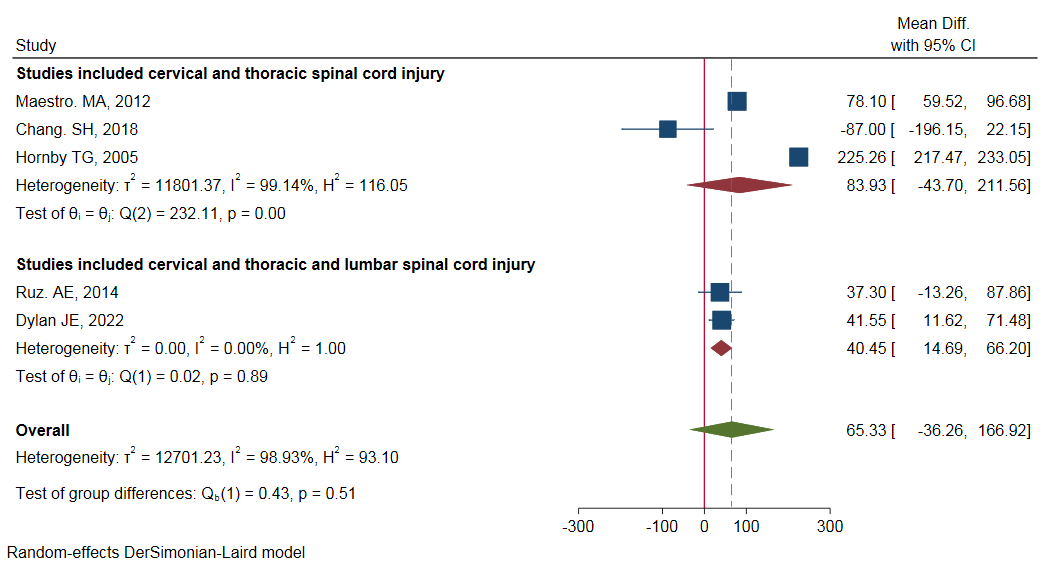


**Fig. S8** Subgroup analysis of the level of spinal cord injury between robotic assisted gait training versus conventional physical therapy on distance of walking (m) in patients with incomplete spinal cord injury

**
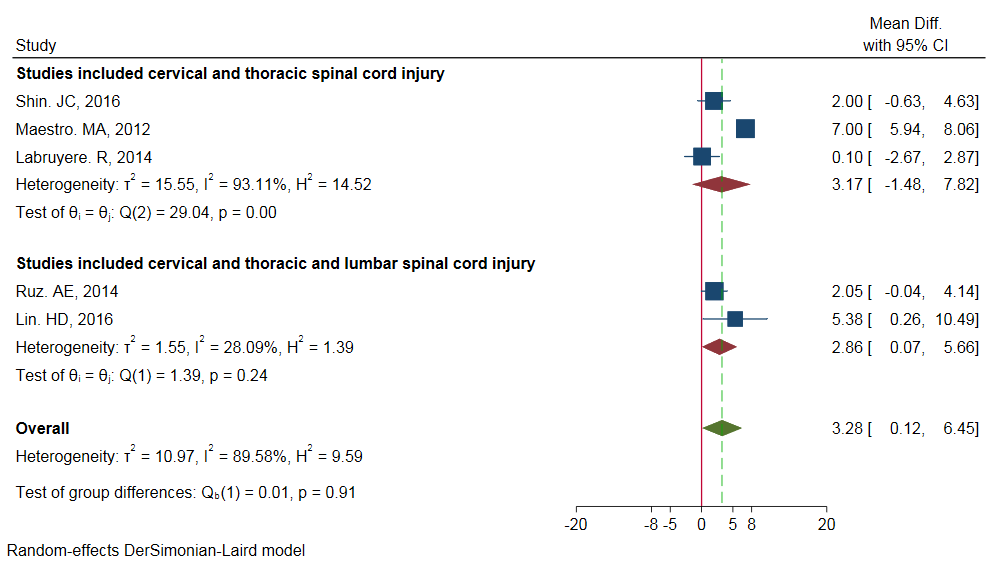
**

**Fig. S9** Subgroup analysis of the level of spinal cord injury between robotic assisted gait training versus conventional physical therapy on Walking Index Spinal Cord injury (WISCI) outcome in patients with incomplete spinal cord injury

**
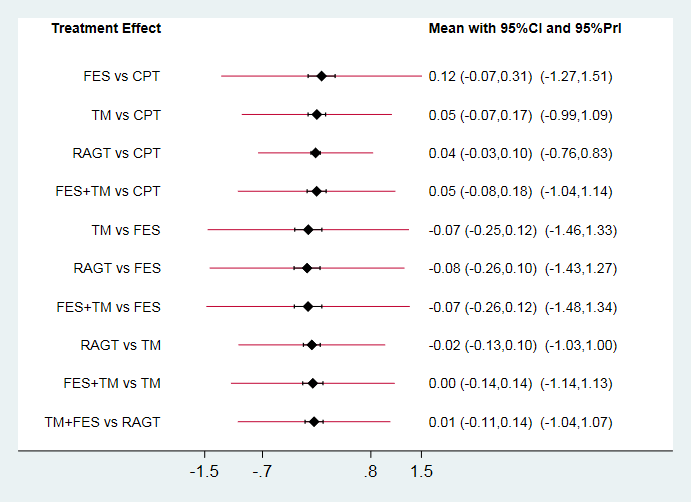
**

**Fig. S10** Predictive interval plots of mean difference for velocity (m/s) of 10 pairwise comparisons estimated; values in right column showed mean difference (95% confidence interval) (95% predictive interval)


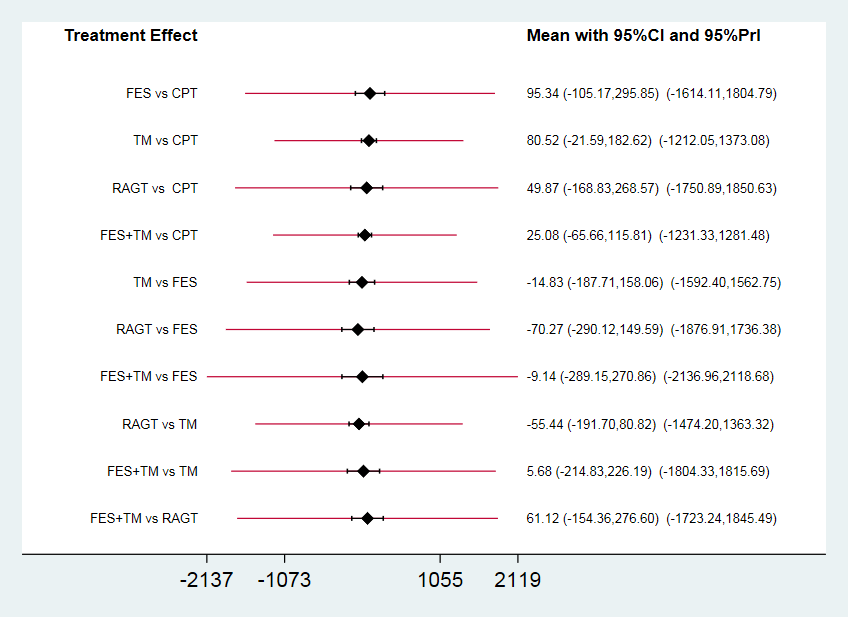


**Fig.S11** Predictive interval plots of mean difference for distance (m) of 10 pairwise comparisons estimated; Values in right column showed mean difference (95% confidence interval) (95% predictive interval)
